# Angioedema and Fatty Acids

**DOI:** 10.3390/ijms22169000

**Published:** 2021-08-20

**Authors:** Akane Wada, Yu Sawada, Hitomi Sugino, Motonobu Nakamura

**Affiliations:** Department of Dermatology, Faculty of Medicine, University of Occupational and Environmental Health, 1-1, Iseigaoka, Yahatanisi-Ku, Kitakyushu, Fukuoka 807-8555, Japan; akane-derma@med.uoeh-u.ac.jp (A.W.); hsugino@med.uoeh-u.ac.jp (H.S.); motonaka@med.uoeh-u.ac.jp (M.N.)

**Keywords:** angioedema, fatty acids, hereditary angioedema

## Abstract

Angioedema is a life-threatening emergency event that is associated with bradykinin and histamine-mediated cascades. Although bradykinin-mediated angioedema currently has specific therapeutic options, angioedema is sometimes intractable with current treatments, especially histamine-mediated angioedema, suggesting that some other mediators might contribute to the development of angioedema. Fatty acids are an essential fuel and cell component, and act as a mediator in physiological and pathological human diseases. Recent updates of studies revealed that these fatty acids are involved in vascular permeability and vasodilation, in addition to bradykinin and histamine-mediated reactions. This review summarizes each fatty acid’s function and the specific receptor signaling responses in blood vessels, and focuses on the possible pathogenetic role of fatty acids in angioedema.

## 1. Introduction

The human body is surrounded by various external environments and is continually exposed to various harmful substances [1,2,3]. As a protective function of the human body against these environmental stimuli, the epithelial immune reaction attempts to remove these dangerous factors. A representative host immune protective reaction is edema, which plays a cleansing function in the epithelial host defense after the entry of external environmental factors [4]. For instance, an insect bite causes the entry of external substances from the insect’s saliva, and causes rapid cutaneous responses by forming a wheal reaction in several minutes and resolution within 24 h. The localized edematous reaction is a biological defense mechanism that dilutes external toxins and pushes them to the outermost layer of the human body. On the other hand, this edema reaction sometimes exacerbates the inflammatory reaction and unexpectedly leads to life-threatening events. This disease is angioedema.

The current advancement of research regarding angioedema has identified its detailed molecular mechanism. Hereditary angioedema, which is one of the forms of angioedema, is closely associated with bradykinin-mediated pathogenesis due to insufficient production of the C1 inhibitor. C1-inhibitor supplementation and icatibant obtain a therapeutic clinical effect for hereditary angioedema [5,6]. On the other hand, histaminergic action has also been postulated as the pathogenesis of nonhereditary angioedema. However, antihistamine agents have not obtained a satisfactory level in clinical outcome in patients with angioedema, suggesting that other molecular mechanisms might also contribute to the development of angioedema.

Fatty acids are essential components for the human body that establish cell membranes, and interact with some physiological and pathological reactions in the human body as mediators [7]. Prostaglandins and leukotrienes have specific receptors that show different pharmacological actions. Therefore, receptor-specific action is helpful to obtain a better understanding of fatty-acid-mediated physiological and pathological responses. Since cyclo-oxygenase inhibitors are the trigger for angioedema [8], it is assumed that fatty acids might also have a bifunctional action in both beneficial and unbeneficial effects on angioedema. However, little is known about the actual relationship between angioedema and fatty acids. In this review, we focus on the role of prostanoids in the pathogenesis of angioedema, and on an update of the knowledge gained from current research.

## 2. Angioedema Pathogenesis

Angioedema influences various superficial human body organs, such as the skin and mucosal membranes, gut, and larynx, and causes acute-response edema by vascular leakage in the deep vessels. The targeted blood vessels are in the deep dermis or subcutaneous tissue [9]. Upon disease onset, angioedema rapidly develops over several minutes to hours [10]. Laryngeal edema is a representative life-threatening event and can require emergency therapy, including intubation. In addition, angioedema in the gut worsens abdominal pains and sometimes causes intussusception, which can also require emergency surgical treatment.

There are several subtypes of angioedema which depend on key molecules to cause angioedema and the underlying-mechanism-involved substances, such as histamine or bradykinin. There are different subtypes of angioedema as per the European Academy of Allergy and Clinical Immunology (EAACI) guidelines [11]. Bradykinin-induced angioedema (C1-INH deficiency/defect or C1-INH normal), mast-cell-mediated angioedema (IgE-mediated or non-IgE mediated angioedema), and angioedema with an unknown mediator (idiopathic angioedema) are representative major subtypes of angioedema. Bradykinin-induced angioedema with C1-INH deficiency/defect is classified into inherited and noninherited angioedema; HAE I and II are inherited angioedema, and acquired angioedema is noninherited. Mast-cell-mediated angioedema is classified into IgE-mediated and non-IgE-mediated angioedema. IgE-mediated angioedema is angioedema with urticaria and anaphylaxis, and non-IgE-mediated angioedema [12] is angioedema with urticaria.

In mast-cell-induced angioedema, IgE-mediated angioedema is closely associated with a histamine-mediated mechanism, which is a Type I allergic reaction to drugs and external allergens [13,14]. ACE inhibitor is the major representative trigger of non-IgE-mediated angioedema. On the other hand, bradykinin-induced angioedema causes C1 esterase inhibitor deficiency due to an inherited gene issue [15]. In some cases, angiotensin-converting enzyme agents also contribute to the development of bradykinin-mediated angioedema [16], in addition to estrogen-mediated effects of the ACE suppressive function [17].

### 2.1. Bradykinin-Mediated Mechanism

**Figure 1:**
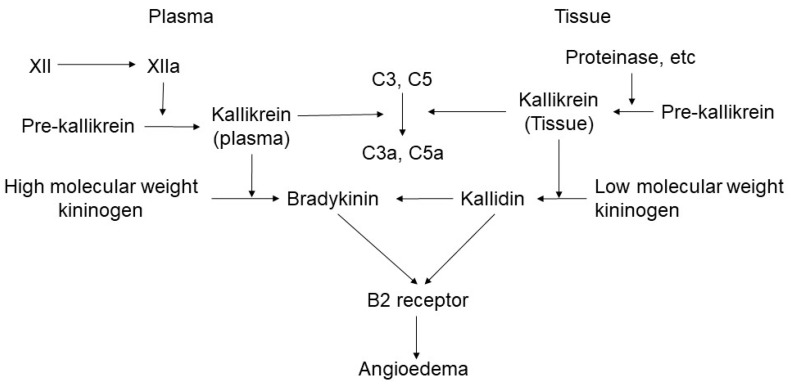
Cascade of bradykinin-mediated angioedema. Bradykinin is produced in the process of activating the kinin–kallikrein system and vascular-coagulation system cascade.

Hereditary angioedema is a type of bradykinin-mediated angioedema. Three types of hereditary angioedema have been reported. Hereditary angioedema Types I and II are caused by a mutation in the C1 esterase inhibitor gene (SERPING1). HAE-3 is associated with mutations of the factor XII (FXII-HAE) gene [11]. Recently, two new mutations in angiopoietin-1 (ANGPT1) and plasminogen (PLG) were reported [11]. Acquired angioedema C1 inhibitor insufficiency is also recognized in patients with angioedema due to C1-INH deficiency on an acquired basis [18]. C1 esterase inhibitor suppresses bradykinin cascade (Figure 1); therefore, its deficiency results in the activation of bradykinin-mediated angioedema, leading to the enhancement of kallikrein production from prokallikrein. Kallikrein enhances the production of bradykinin from high-molecular-weight kininogen.

In addition, tissue kallikrein is involved in the pathogenesis of angioedema. Tissue prekallikrein is processed into kallikrein by proteinases in the tissue and enhances the production of kallidin, which is an upstream material of bradykinin, acts on B2 receptor, and enhances the pathogenesis of angioedema.

Bradykinin has been recognized in two specific receptors, B1 and B2. Bradykinin binds to the B1 receptor, leading to increased intracellular calcium ion influx and the enhancement of inflammatory reactions. Bradykinin also binds to the B2 receptor, which is responsible for the pathogenesis of angioedema. The B2 receptor belongs to a G protein-coupled receptor coupled to Gq and Gi. Gq stimulates phospholipase C activation to enhance intracellular calcium influx, and Gi suppresses adenylate cyclase. The B2 receptor also has a unique protein complex with angiotensin-converting enzymes (ACE), suggesting that this complex protein formation plays an important role in the interplay mechanism between the renin–angiotensin and kinin–kallikrein systems. Bradykinin binds to the B2 receptor, and rapidly causes vasodilation and vascular permeability.

There is no evidence that bradykinin itself enhances the release of histamine from mast cells and forms wheal, indicating that bradykinin-mediated hereditary angioedema basically does not cause urticaria, which is a helpful objective symptom to distinguish nonbradykinin-mediated angioedema from bradykinin-mediated angioedema.

### 2.2. Histamine-Mediated Mechanism as IgE-Mediated Angioedema

Histamine is a representative driver that causes urticaria and the enhancement of vascular permeability leading to angioedema. One of the clinical differential diagnostic symptoms is the presence of urticaria in histamine-mediated angioedema, because the action point is different between bradykinin- and histamine-mediated angioedema.

Histamine plays important physiological roles mediated through three histamine receptors, H1, H2 and H3, which belong to G protein-coupled receptors [19]. The H1 receptor is a representative histamine receptor that causes urticaria, and mediates wheal formation and vascular permeability [20,21]. Histamine is a relevant vasoactive derived from mast-cell degranulation, and the H1 receptor leads to vasodilatation and enhances blood flow [22]. The H2 receptor is also involved in histamine-related vascular permeability [23,24]. In intestinal vascular permeability, the H1 receptor antagonist does not alter histamine-induced vascular permeability, but it blocks the initial vasodilation. The H2 receptor antagonist significantly reduces histamine-induced vascular permeability while showing a slight effect on initial vasodilation [25]. Although H3 inhibition alone does not have that effect, the combination of an H1 antihistamine agent with an H3 antagonist shows a synergy effect on urticaria [26].

### 2.3. Medical-Agent-Mediated Angioedema as Non-IgE Mediated Angioedema

Many unknown triggers are recognized; however, medications are well-known as a trigger of angioedema. ACE is a representative agent that causes angioedema. ACE escalates the metabolization of bradykinin. On the other hand, ACE inhibitors act as a suppressor for bradykinin metabolism and increase the serum bradykinin level leading to the enhancement of angioedema pathogenesis. Although bradykinin-targeted therapy is useful for hereditary angioedema, it might also be useful for the treatment of drug-induced angioedema. Icatibant is effective for ACE inhibitor-triggered angioedema, which is also involved in the enhancement of bradykinin production, suggesting that treatment based on etiology is essential for the treatment of angioedema [27].

Estrogen agents, especially pills, are also a trigger of angioedema. Estrogen plays a suppressive effect on ACE, resulting in the same pathway of ACE inhibitor-induced angioedema. In that case, danazol is a candidate therapeutic agent for angioedema.

### 2.4. Recent Advancement of Angioedema Treatment

The plasminogen system, the contact system that generates bradykinin, and the complement system are connected in the underlying pathogenesis of angioedema [28]. Plasmin is also able to activate the classical pathway for the generation of C3a and C5a, and subsequently induces degranulation in mast cells. A recently updated understanding of angioedema helped to develop novel therapeutic options, especially lanadelumab and berotralstat, which target the upstream factors, accelerating bradykinin production. Lanadelumab is a human-specific monoclonal antibody targeting plasma kallikrein that acts as an inhibitor and reduces downstream bradykinin generation; several studies showed that lanadelumab potentiates the therapeutic preventive effect for angioedema development [29,30,31,32]. Berotralstat suppresses the development of angioedema by the inhibitory action of plasma kallikrein, subsequently decreases bradykinin production, and shows strong regulatory action for angioedema; this efficacy was confirmed by several clinical studies [33,34,35,36]. These agents are expected to broaden the range of treatment options for angioedema and improve clinical outcomes.

## 3. Prostanoids and Their Metabolisms

Fatty acids consist of a carboxylic acid with a long aliphatic chain, and they are important dietary sources of fuel and structural components for cells [7]. Arachidonic acid is released from the cell membrane by phospholipase A2 (PLA2), and is then converted into prostaglandins and thromboxane by cyclooxygenase (COX), in addition to leukotrienes (LT) by lipoxygenase (LOX); these metabolites show various physiological activities depending on each specific receptor’s signaling.

As with the other fatty acids, omega-3 fatty acids are classified into three lipids: α-linoleic acid (ALA), docosahexaenoic acids (DHA), and eicosapentaenoic acid (EPA). ALA is changed into EPA and subsequently converted into DHA by enzymatic activity [7]. Although these enzymatic conversions occur in the liver, these enzymes are extremely limited in the human body. Therefore, we need to take in these omega-3 fatty acids from enriched foods or supplementation. Omega-3 fatty acids derived from fish oil and nuts show a beneficial impact on human diseases via anti-inflammatory action. Recent studies also showed that the metabolites of omega-3 fatty acids have strong anti-inflammatory action [37,38,39].

In addition to these representative fatty acids, commensal bacteria in the human body also contribute to generating short-chain fatty acids, such as butyrate and propionate. Commensal bacteria have the potential to metabolize dietary fiber under anaerobic conditions, and generate short-chain fatty acids. This generation is observed in the gut and hair follicles in the skin. These fatty acids are helpful for human cells in the nutrition of epithelial-cell growth. In addition to the physiological function, short-chain fatty acids influence gene transcription, mediated by epigenetic modification. This epigenetic change regulates transient gene transcription by the mechanism of histone acetylation.

## 4. Evidence of Prostanoid-Related Angioedema

### 4.1. COX-Related Angioedema

To clarify a possible role of prostanoids in the pathogenesis of angioedema, there are reports regarding COX inhibitor-related angioedema. Nonsteroidal anti-inflammatory drugs (NSAIDs) are one of the triggers that enhance edema in patients with angioedema [8]. For instance, COX impacts blood-vessel barrier dysfunction, mediated by the impairment of occludin [40]. Bradykinin-mediated angioedema is suppressed by COX inhibitors [41], suggesting that the impact of prostanoids in angioedema is different between bradykinin- and nonbradykinin-mediated angioedema. As another aspect of the contribution of COX in the development of angioedema, COX-mediated metabolite prostanoids might also play some role in the pathogenesis. The arachidonic cascade begins from COX metabolic action, and subsequently releases PGD2, PGE2, PGF2, PGI2 and TxA2. These prostanoids play various roles in physiological and pathological action in skin diseases [42,43].

#### 4.1.1. PGE2 and Angioedema

PGE2 itself has beneficial potential for angioedema and prevents vascular permeability [44,45]. PGE2 has four specific receptors, EP1, EP2, EP3 and EP4; each receptor shows unique reactions in vascular permeability and blood-vessel dilation, and sometimes shows different reactions depending on each receptor’s response. In addition, EP2 or EP4 receptor agonists promote vascular permeability by the enhancement of peripheral vessel dilation and local blood flow [46]. On the other hand, EP3 receptor agonists suppress vascular permeability without altering vascular diameter and blood flow [45]. The EP1 signal also has a unique effect on vascular barrier function, and contributes to the disruption of blood–brain barrier function [47], leading to the enhancement of vascular permeability.

PGE2 might be associated with histamine-mediated angioedema, and it involves the regulation of mast-cell degranulation. EP2 signaling inhibits mast-cell degranulation and migration [48,49]. EP3 signaling elicits histamine release in mouse peritoneal and bone-marrow-derived mast cells [50]. PGE2 potentiates the IgE-mediated histamine release from cultured mast cells via EP3 and/or EP1 receptors [51].

Contrary to histamine-mediated angioedema, PGE2 enhances bradykinin-induced hyperalgesia, whereas it has no effect on edema [52], suggesting that PGE2 might also play a role in the pathogenesis of bradykinin-mediated angioedema in some cases.

#### 4.1.2. PGD2 and Angioedema

Since PGD2 is involved in the pathogenesis of allergic diseases, PGD2 is also expected to involve the pathogenesis of angioedema. As potent histamine-mediated angioedema, PGD2 production is observed in mastocytoma [53], cold urticaria [54], and localized heat urticaria [55]. As expected, the antihistamine agent suppresses the potent IgE-mediated production of histamine and PGD2 [56]. These findings suggest that PGD2 boosts histamine-mediated cutaneous reaction, and antihistamine agents are expected to regulate PGD2-mediated reaction in angioedema. On the other hand, PGD2 itself suppresses histamine production, and PGD2 inhibits IgE-mediated scratching by suppressing histamine release from mast cells [57]. In addition, PGD2 receptor CRTH2-466T>C gene polymorphism contributes to the required dose of antihistamines in patients with chronic urticaria [58], suggesting that PGD2 might be a negative regulator in the pathogenesis of histamine-mediated urticaria reaction in the skin.

The potency of PGD2 in vascular permeability seems to be different depending on the pathogenesis of disease onset. PGD2 enhances the endothelial barrier through the DP receptor in inflamed tissue [59,60]. In addition, in vitro experiments showed that DP agonism enhances vascular endothelial barrier formation [61] via the cAMP/PKA/Tiam1/Rac1 pathway [62]. On the other hand, mast-cell-derived PGD2 acts as a negative regulator of vascular permeability and systemic symptoms in a murine model of anaphylaxis in vivo [63]. These findings suggest that PGD2 might have bifunctional effects on vascular permeability in the presence or absence of histamine. Consistently, PGD2 enhances bradykinin-induced hyperalgesia and edema [52], suggesting that PGD2 is at least a positive driver in the pathogenesis of bradykinin-mediated angioedema.

#### 4.1.3. PGF2 and Angioedema

PGF2α is produced in various allergic diseases [64,65], and PGF2α is a biomarker for mast-cell disorders [66]. Although there is no report regarding PGF2-related angioedema, PGF2 increases vascular permeability to enhance protein leakage [67], and histamine promotes PGF2 production [68], suggesting that PGF2 might also be involved in the pathogenesis of angioedema.

#### 4.1.4. PGI2 and Angioedema

PGI2 is produced in allergic diseases such as anaphylaxis [69]. PGI2 acts as a vasodilator [70], and it enhances the increased vascular permeability induced by bradykinin and histamine [71]. Bradykinin promotes PGI2 synthesis [72]; however, PGI2 itself is ineffective on histamine release [73]. Therefore, PGI2 might be more potent in enhancing angioedema.

#### 4.1.5. Thromboxane A2 (TxA2) and Angioedema

TxA2 is generated from PGH2 downstream of arachidonic acids mediated by thromboxane-A synthase in a metabolic reaction, and enhances various inflammatory skin diseases. TxA2 is released from mast cells [74], and histamine enhances TxA2 production [75]. Thromboxane A2 contributes to increased permeability after thrombin, since the inhibition of thromboxane synthesis prevents permeability changes [76,77,78]. TxA2 also enhances IL-8 production, leading to the enhancement of vascular permeability [79].

#### 4.1.6. Phospholipase A2 (PLA2) and Angioedema

A recent study found an interesting novel role of phospholipase A2 (PLA2) in the pathogenesis of angioedema [80]. PLA2 is a representative enzyme that hydrolyzes the fatty acid from membrane glycerophospholipids, releasing arachidonic acid and lysophospholipids. Secreted or extracellular PLA2 directly modulates endothelial-cell migration and vascular permeability, indicating that PLA2 plays an essential role in the development of hereditary angioedema with C1-inhibitor deficiency. Furthermore, the overall genetic variation in the PLA2 gene (PLA2G4A) in NSAID-induced angioedema was assessed, and rs2049963 was strongly associated with an increased risk for NSAID-induced angioedema [81], suggesting that PLA2 might be more widely involved in the pathogenesis of angioedema than was previously thought.

### 4.2. Leukotrienes

Leukotrienes are derived from arachidonic acids mediated by arachidonate 5-lipoxygenase [82]. ALOX5 converts arachidonic acid into 5-hydroperoxy eicosatetraenoic acid (5-HPETE), and subsequently into leukotriene A4 (LTA4). LTA hydrolase converts LTA4 into LTB4, which is a proinflammatory and chemoattractive agent. In addition, LTC4 synthase accelerates the LTA4 metabolite into LTC4, and is subsequently converted into LTD4 and LTE4 by ubiquitous enzymes. These leukotrienes are involved in the pathogenesis of various allergic diseases.

#### 4.2.1. LTB4 and Angioedema

LTB4 is a representative inflammatory lipid mediator which enhances inflammatory reactions in various human diseases [38]. LTB4 is metabolized from arachidonic acid mediated by ALOX-5. LTB4 increases vascular permeability [83,84]. Intradermally injected LTB4 elicits a transient wheal and flare [85]. LTB4 has two specific receptors, BLT1 and BLT2, and BLT1 inhibitor CP 105,696 suppresses vascular permeability [86,87]. BLT2 deficiency enhances vascular permeability in inflamed tissue [88].

LTB4 prolongs vasodilation reaction in the skin [89], which is not altered by NSAID administration [89], suggesting that LTB4 independently contributes to the development of vasodilation.

LTB4 enhances histamine production [90], and bradykinin enhances LTB4 production [91], suggesting that LTB4 might play a role in the cross-counter between histamine- and bradykinin-mediated angioedema.

#### 4.2.2. LTC4 and Angioedema

LTC4 is a metabolite from LTA4 that is a positive driver of allergic diseases [92]. LTC4 has the potential to enhance vascular permeability [84,93]. LTC4 enhances increased vascular permeability [94]. LTC4-induced skin reactions in the mouse ear were significantly suppressed by the administration of chlorpheniramine [95], while the effect of LTC4 on the vasodilation is controversial [94,96].

#### 4.2.3. LTD4 and Angioedema

LTD4 is an allergic driver in various diseases, and it enhances vascular permeability [93] and vasodilator responses [96,97]. Since there is no direct evidence of the synergistic relationship between bradykinin or histamine and LTD4, there might be only a limited role of LTD4 in the pathogenesis of angioedema.

### 4.3. PUFA and Angioedema

Omega-3 polyunsaturated fatty acids are derived from fish oil and nuts, and have potent anti-inflammatory action in various human diseases. Representative omega-3 fatty acids are EPA and DHA; both these omega-3 fatty acids and their metabolites also show strong anti-inflammatory action. Polyunsaturated fatty acids suppress PGE2 production by mast cells [98,99], and contribute to the suppression of angioedema development. Omega-3 fatty acid metabolites also suppress mast-cell function. Maresin-1, resolvin E1 (RvE1), and RvD1 suppress mast-cell infiltration into the skin [100,101,102]. RvD1 impairs capillary permeability after inflammatory reaction [103], preventing edema in the tissue. These functions might beneficially impact the suppression of angioedema. Since there is no clinical research investigating the actual impact of omega-3 fatty acids to prevent the development of angioedema, further investigations are needed to clarify the effect of omega-3 fatty acids.

### 4.4. Short-Chain Fatty Acid and Angioedema

Short-chain fatty acids, such as butyrate and propionate, are derived from bacteria under anaerobic conditions, and can modulate various physiological and pathological actions in human organs [104,105]. In the gut, dietary fibers are metabolized into short-chain fatty acids by gut microbiomes, and show various benefits on health [106]. Recent studies have also revealed that short-chain fatty acids influence epigenetic modification and regulate gene expression.

DNA sequencing information does not basically reveal major genetic changes; however, environmental factors affect gene expression by chemical epigenetic modifications, which influence DNA itself and DNA-binding proteins, especially through histone modification, leading to chromatin-structure remodeling and altered gene transcription.

Histone is positively charged due to the presence of lysine and arginine amino acids in histone tails. This characteristic of histone supports binding to negatively charged DNA. The acetylation of histone cancels the positive charges of histone by the chemical modification of amino acid residues from histone, and impairs the binding affinity to DNA, leading to chromatin structure changes; it influences gene transcription by making an open chromatin site. Deacetylated histone can tightly connect to DNA to prevent gene transcription, while histone acetylation can release the tight connection, and make it easy to conduct gene transcription. Histone acetylation is regulated by histone acetyltransferases (HATs) and histone deacetylases (HDACs). Short-chain fatty acids derived from microorganisms act as HDAC inhibitors and show diverse gene transcription.

Low concentrations of butyrate and propionate decrease permeability without inducing cell damage [107]. HDAC3 inhibition protects transendothelial-cell permeability and the downregulation of tight-junction protein claudin-5 [108]. In addition, HDAC9 contributes to oxygen-glucose deprivation-induced brain-microvessel endothelial-cell dysfunction, showing permeability dysfunction accompanied by the reduced expression of tight-junction proteins [109]. HDAC9-silencing endothelial cells promote recovering damaged tight-junction proteins, ZO-1, occludin, and claudin-5 [109]. Therefore, short-chain fatty acids can suppress vascular permeability depending on HDAC3 and HDAC9 suppression.

## 5. Therapeutic Potential for Fatty Acids Involving Angioedema

We summarized the detailed actions of fatty acid-mediated angioedema and the possible therapeutic potential of fatty acids for angioedema. In COX-mediated fatty acids, PGE2-EP3 signaling and PGD2 in bradykinin-mediated angioedema are only a negative regulator for angioedema, suggesting that COX inhibitors might influence pathogenesis mediated by this signaling.

In PGE2, antagonists against EP1, EP2, and EP4 might have therapeutic potential to suppress the development of angioedema by the suppression of vascular permeability. PGE2 only has a beneficial effect mediated by EP3 signaling; the EP3 agonist can enhance vascular permeability and might be a therapeutic candidate for angioedema treatment. PGD2 signaling suppression by a DP antagonist impairs vascular permeability in bradykinin-mediated angioedema, and is a therapeutic target for angioedema. However, it remains unclear which PGD2 receptors are responsible for the pathogenesis of angioedema. Histamine-mediated PGD2 has the opposite effect on vascular permeability. Because PGF2, PGI2, and TxA2 act for the development of vascular permeability, this signaling suppression might be a therapeutic candidate for angioedema.

Contrary to COX-mediated fatty acids, all LTs enhance vascular permeability, which contributes to the development of angioedema. Therefore, the suppression of LT synthesis and receptor blockades is a candidate for the treatment of angioedema. In LTB4, BLT1 signaling blockages can suppress the development of angioedema; it is commercially available and currently used for the treatment of allergic diseases [110,111].

Since omega-3 fatty acids show some beneficial impact on angioedema, DHA, EPA, and their metabolites are therapeutic candidates for the treatment of angioedema. To clarify the actual impact of omega-3 fatty acids, a larger clinical study is needed.

Microbiome-derived short-chain fatty acids have beneficial potential for the suppression of angioedema. Although the actual relationship between the microbiome in the gut and the pathogenesis of angioedema remains unclear, dietary fiber intake might have beneficial potential for angioedema by driving short-chain fatty acid production in the body. To obtain a more specific effect of short-chain fatty acids, HDAC3 or HDAC9 specific inhibitors might show inhibitory effects on angioedema development. Although there is no specific inhibitor for HDAC3 and HDAC9, and almost all HDAC inhibitors have multitargeted HDAC suppression, vorinostat and entinostat have HDAC3 suppressive effects [112,113]. Although there is no clinically available HDAC9 inhibitor, TMP269 shows HDAC9 suppressive function.

## 6. Conclusions

Our review introduced the possible involvement of fatty acids in the pathogenesis of angioedema. Fatty acids exhibit various specific receptors that show different actions from the same ligand action. Understanding such actions of the fatty acids themselves is therefore helpful to obtain a better understanding of fatty-acid-mediated angioedema, in addition to each receptor-mediated reaction. Although there are a limited number of studies regarding angioedema and epigenetic modification, gut-microbiome analysis might be helpful to uncover the underlying mechanisms of angioedema. The contribution of fatty acids may not be the main cause of the pathogenesis of angioedema, but this contribution is not small and may be a therapeutic target for the treatment of angioedema.

## Data Availability

Not applicable.
